# Novel technique of additional anchor plastic stent placement during endoscopic ultrasound-guided gallbladder drainage

**DOI:** 10.1055/a-2277-0615

**Published:** 2024-03-20

**Authors:** Toji Murabayashi, Ryutaro Matsushima, Shinya Sugimoto

**Affiliations:** 1 37071 Department of Gastroenterology, Ise Red Cross Hospital, Ise, Japan

A fully covered self-expanding metal stent (FCSEMS) is frequently used for endoscopic ultrasound-guided gallbladder drainage (EUS-GBD) in some countries where the use of lumen-apposing metal stent for EUS-GBD is not approved 1 2 . Coaxial placement of a double-pigtail plastic stent (DPPS) as an anchor within the FCSEMS for EUS-GBD is preferred to prevent food impaction, delayed bleeding, and stent migration 2 3 . However, placing the additional stent through the distal end of the FCSEMS can be challenging and risky, as the pushing force of the DPPS may be transmitted to an inappropriate axis on the bent portion of the long FCSEMS ( Fig. 1 **a** ). We developed a novel technique to overcome these technical difficulties ( Fig. 1 **b** ).

**Fig. 1:**
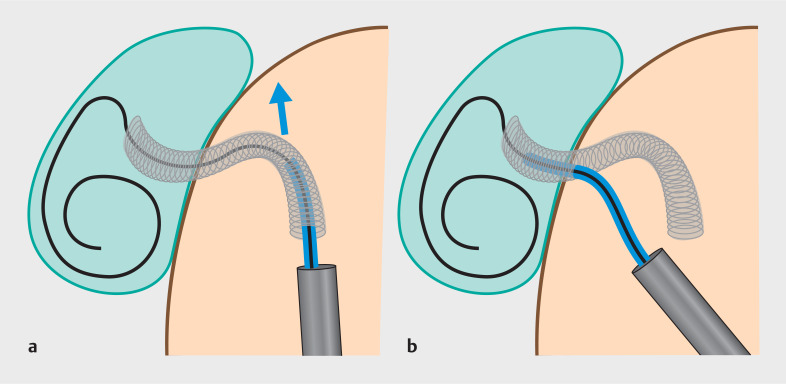
Schematic depicting the technical challenges associated with additional stent placement as an anchor and the novel technique (catheter-puncture method) employed during endoscopic ultrasound-guided gallbladder drainage (EUS-GBD). **a** In cases where the long portion of the fully covered self-expandable metal stent (FCSEMS) is located in the duodenum, additional stent placement through the distal end of the FCSEMS is challenging as the pushing force of stent delivery may be transmitted to an inappropriate axis on the bent portion of the FCSEMS. **b** A schematic depicting the novel technique of additional anchor plastic stent placement during EUS-GBD (catheter-puncture method). The approach from the side of the FCSEMS near the duodenal wall significantly facilitates additional stent insertion. This is attributed to the direct transmission of the pushing force of the stent delivery to the appropriate axis.

A 75-year-old woman with unresectable distal bile duct cancer who had previously undergone FCSEMS placement in the bile duct underwent EUS-GBD for acute cholecystitis ( Fig. 2 ). Following puncture with a 19-gauge needle and dilation with a 4-mm balloon dilator, an FCSEMS (HANAROSTENT Benefit, 8 × 100 mm) was deployed from the gallbladder to the duodenum. As 6 cm of the FCSEMS was positioned in the duodenum, additional placement of a plastic stent through the distal end of the FCSEMS appeared to be technically challenging. Therefore, we placed a plastic stent using a novel technique ( Video 1 , Fig. 3 ). A standard catheter and guidewire were inserted into the FCSEMS and gallbladder from the side of the FCSEMS near the duodenal wall after puncturing the membrane of the FCSEMS using the catheter under direct endoscopic visualization. A DPPS (7-Fr × 7 cm) was inserted into the gallbladder through the guidewire without dilation. It was positioned as a bridge connecting the gallbladder, FCSEMS, and duodenum in a lambda-shaped configuration.

**Fig. 2:**
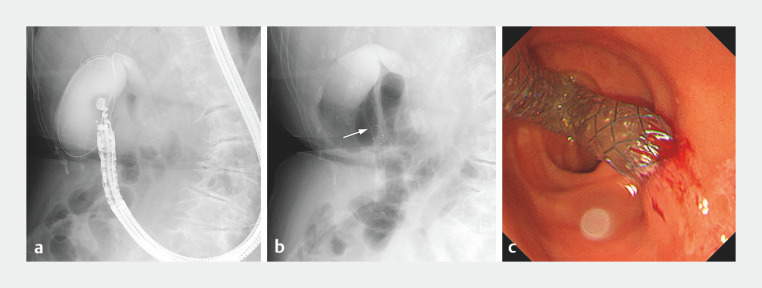
Fluoroscopic and endoscopic images during EUS-GBD. **a** Fluoroscopic image depicting the gallbladder filled with contrast medium and a guidewire inserted into the gallbladder from the duodenal wall. **b** Fluoroscopic image showing an FCSEMS positioned from the gallbladder to the duodenum. Six centimeters of the FCSEMS are located in the duodenum. The arrow indicates the duodenal wall. **c** Endoscopic image showing FCSEMS placed on the duodenal wall. The duodenal portion of FCSEMS is elongated and exhibits a bent configuration.

**Fig. 3:**
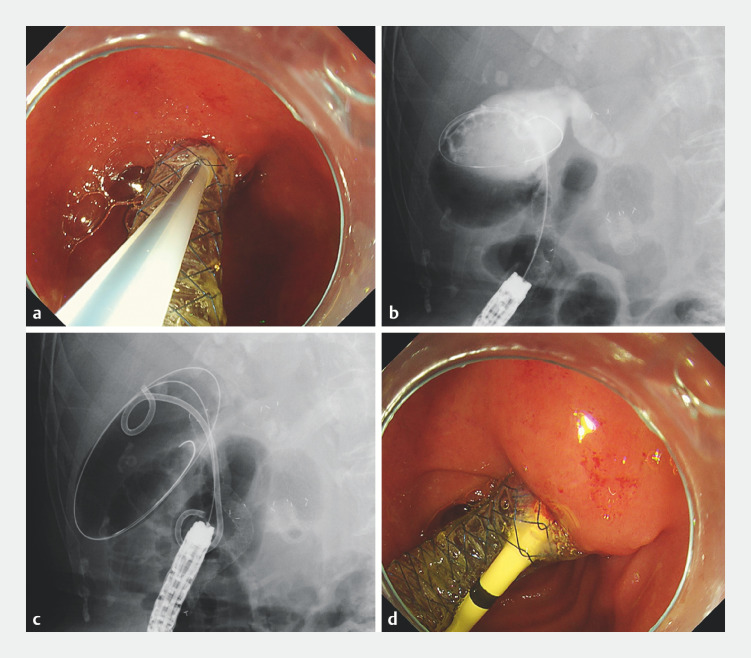
Endoscopic and fluoroscopic images during additional placement of a double-pigtail plastic stent (DPPS). **a** Endoscopic image showing a catheter puncturing the FCSEMS from the side near the duodenal wall. **b** Fluoroscopic image showing a guidewire inserted into the FCSEMS and the gallbladder from the side of the FCSEMS near the duodenal wall. **c** Fluoroscopic image showing the DPPS placed from the side of the FCSEMS near the duodenal wall into the FCSEMS and gallbladder, bridging the gallbladder, FCSEMS, and duodenum in a lambda-shaped configuration. A second guidewire is inserted along the DPPS. **d** Endoscopic image showing the DPPS placed from the side of the FCSEMS near the duodenal wall into the FCSEMS and gallbladder.

A novel technique for an additional anchor plastic stent placement during endoscopic ultrasound-guided gallbladder drainage.Video 1

Using this novel technique, additional DPPSs can be readily placed, even in cases where an exceptionally long portion of FCSEMS is located in the duodenum.

Endoscopy_UCTN_Code_TTT_1AS_2AH

## Notes

Endoscopy E-Videos https://eref.thieme.de/e-videos
*E-Videos* is an open access online section of the journal *Endoscopy* , reporting on interesting cases and new techniques in gastroenterological endoscopy. All papers include a high-quality video and are published with a Creative Commons CC-BY license. Endoscopy E-Videos qualify for HINARI discounts and waivers and eligibility is automatically checked during the submission process. We grant 100% waivers to articles whose corresponding authors are based in Group A countries and 50% waivers to those who are based in Group B countries as classified by Research4Life (see: https://www.research4life.org/access/eligibility/ ). This section has its own submission website at https://mc.manuscriptcentral.com/e-videos .
